# Life Expectancy of Persons with Disability in Italy: Estimation Based on an Administrative Cohort from 1999 to 2012

**DOI:** 10.3390/epidemiologia7030065

**Published:** 2026-05-07

**Authors:** Aldo Rosano, Alessandro Solipaca, Luisa Frova, Gabriella Sebastiani, Paola Di Filippo, Stefano Marchetti, Lucilla Scarnicchia

**Affiliations:** 1 National Institute for the Analysis of Public Policies (INAPP), 001980 Rome, Italy; 2 National Institute of Statistics (ISTAT), 00198 Rome, Italy; solipaca@istat.it (A.S.); frova@istat.it (L.F.); gab.sebastiani@gmail.com (G.S.); difilippo@istat.it (P.D.F.); stmarche@istat.it (S.M.); lucilla.scarnicchia@istat.it (L.S.)

**Keywords:** survival, disability, life expectancy, policy implications

## Abstract

Background: Estimates of life expectancy of people with disabilities are scarce and tend to focus on individuals with specific diseases. The health conditions of people with disabilities are often very poor, which is reflected in their significantly lower life expectancy compared to the rest of the population. Objectives: This study aims to estimate the life expectancy of people with severe disabilities in Italy. Methods: Data from the 1999–2000 Health Interview Survey, linked to the register of causes of death up to 2012, were utilized for the purpose of this study. Survival was analyzed using a Weibull regression model. Hazard ratios for subjects with and without disabilities were employed to estimate the mortality risk among subjects with disabilities compared to all surveyed subjects. These ratios were then employed to construct a life table for people with disabilities by multiplying the death probabilities of the general population by the ratio. Results: The life expectancy at 15 years for people with disabilities was found to be 59.1 years for males and 66.2 years for females. The life expectancy (LE) gap between people with disabilities and the general population at 15 years was 6.6 years for men and 4.1 years for women. Conclusions: These findings provide reliable and robust information on the life expectancy gap between people with and without disabilities and can be used as a reference point when evaluating policies aimed at people with disabilities.

## 1. Introduction

Estimates of the life expectancy (LE) of people with disabilities in Italy are rare and mainly focus on individuals with specific diseases. Even at an international level, survival data concerning specific disability-inducing conditions is available, while estimates concerning disability itself, regardless of cause, are scarce.

Life expectancy estimates for people with disabilities have been conducted using different approaches. One study, conducted in California, analyzed four chronic disability conditions: cerebral palsy, spinal cord injury, Down’s syndrome and intellectual disability (not resulting from Down’s syndrome). The authors estimated life expectancy at 10 years for people with quadriplegia and severe intellectual disability: 40 years for people with quadriplegia, 59 years for people with mild or moderate intellectual disability, and 50 years for people with severe intellectual disability [1]. These estimates are based on cross-sectional studies and, in general, provide more pessimistic results than those from other studies. However, these results are limited to specific pathological conditions. Furthermore, these estimates appear to be applicable to young individuals with severe disabilities that have been present since birth or during the first years of life.

In cases of disability onset in adulthood, a more reliable estimate of survival could be obtained by calculating a penalty coefficient for the various factors causing the disability, and combining this with life expectancy at different ages, as determined from mortality tables for the general population [2].

A study conducted in the Netherlands between 2001 and 2006, in which more than 60,000 respondents completed the ‘Health Module’ of the Ongoing Population Survey (OPS), investigated the survival of people with disabilities. Disability status was defined as an inability to perform at least one of the five activities of daily living (ADLs) ‘normally’ [3]. The eventual deaths of the respondents were verified by linking the OPS data with the municipal population registers as of 31 December 2007. Using a Cox model, the hazard ratio (HR) (the risk of death among people with disabilities compared with people without disabilities) and life expectancy at 55 years, separately for men and women, were then calculated. For men, the HR was 7.8, with a life expectancy at 55 years of 15.9 years. For women, the HR was 6.14, with a life expectancy at 55 years of 21.3 years [4]. A recent Italian study estimated a life expectancy with disability ranging from 8.1 years to 12.6 years in subjects between 50 and 79 years based on a series of longitudinal population surveys [5]. Estimates of LE for people with disabilities can be used as a useful reference in periods that are not affected by exceptional events, such as the 2019–2020 SARS-CoV-2 pandemic, which caused an increase in mortality, particularly among vulnerable individuals, including those with severe disabilities [6].

The study aims to produce reliable estimates of LE for people with severe disabilities in Italy. The study is based on an administrative cohort study, using pre-existing, routinely collected mortality data of the following 13 years, nested in a cross-sectional population survey conducted in 1999–2000. These estimates can still be considered valid references, provided that the age-specific rates of disability and comparative mortality risks between people with and without severe disabilities remain stable.

The study has been conducted following the RECORD protocol for observational studies using routinely collected health data [7].

## 2. Materials and Methods

### 2.1. Data

Data were obtained from the 1999–2000 Health Interview Survey (HIS) and linked to the register of causes of death up to 2012 to determine the vital status of HIS participants. The HIS focuses on key aspects of population health conditions and utilization of health services. Based on a sample of around 52,300 households, the survey was conducted by the Italian National Institute of Statistics (ISTAT) in four waves between September 1999 and March 2000. The Causes of Death Register contains demographic data (e.g., age and sex) which is provided by the Civil Status Officer of the municipality, as well as data on causes of death which is provided by the physician. ISTAT annually collects, processes and publishes data on mortality by cause. The data used for the analyses were derived from a database created using a deterministic linkage procedure between individuals enrolled in the 1999–2000 HIS (140,011 individuals, of whom 128,818 were linkable with the Death Records Archive, equaling 92% of the sample) and the Causes of Death Register for the years 1999–2012. This procedure identified a total of 14,912 deaths.

### 2.2. Methods

#### 2.2.1. Definition of the Population Under Study

Since 1990, ISTAT has employed a set of questions developed by an OECD working group based on the World Health Organization’s International Classification of Impairments, Disabilities and Handicaps to estimate disability prevalence. The questionnaire includes a scale assessing difficulty in performing activities of daily living (ADLs), originally proposed by Katz in the 1960s [8]. In this study, we adopt the OECD-endorsed definition of disability based on Katz [9], which identifies people with severe disabilities with those who are unable to perform at least one of the functions of activities of daily living (ADL) [10]. These activities include walking, climbing stairs, stooping, lying down, sitting, dressing, washing, bathing and eating, as well as sensory functions such as hearing, seeing and speaking. Confinement to bed, a chair (excluding a wheelchair), or home is also considered. This battery has been shown to classify disability severity effectively [11]. The study population therefore comprised individuals who were confined to bed or a chair, or who had at least one severe functional limitation. Disability status was assessed using responses from the 1999–2000 HIS questionnaire. No follow-up assessment of disability status was conducted during the subsequent study period (2000–2012).

#### 2.2.2. Statistical Methods

The methodological approach consisted of four main steps. First, we estimated the transition probability from a healthy state to a disabled state. Second, we calculated age-specific probabilities of death for people with disabilities. Third, we estimated the relative risk of death for individuals with disabilities compared with those without disabilities. Finally, we applied these relative risks to the life table of the general population to estimate life expectancy among people with disabilities.

#### 2.2.3. Estimation of Transition Probabilities

Probabilities of death were calculated by assigning each death occurring during the observation period (1999–2012) to the corresponding age group of the individual at risk. Because disability status was measured only at the time of the 1999–2000 survey, age- and sex-specific transition probabilities from a healthy state to a state of severe disability were estimated to approximate the evolution of disability prevalence during the 13-year follow-up. Although reliable estimation of transition probabilities in multistate health models ideally requires longitudinal data [12], only a limited number of Italian studies have attempted this using longitudinal surveys [5], and these rely on a different definition of disability. In those studies, disability was simplified into a dichotomous variable, irrespective of severity and allowing for recovery—an approach inconsistent with our framework and likely to overestimate transition probabilities. As an alternative, transition probabilities may be inferred from cross-sectional data, which capture health status at a single time point [13,14,15].

In our study, because longitudinal data required for direct estimation of transition probabilities was unavailable, we relied on cross-sectional data. However, such data do not capture individual-level transitions over time, making estimation inherently challenging. Cross-sectional surveys observe different individuals at different points of time rather than following the same individuals over time, which prevents direct observation of transitions between health states [16]. As a result, identification of unobserved transitions is feasible only by imposing assumptions on states and time. Following Rickayzen [17] and Hariyanto [18], we applied a logistic function to estimate one-year transition probabilities across multiple health states, assuming a stationary population structure. Model parameters were calibrated so that the resulting age- and sex-specific prevalence rates closely matched those observed in the 1999–2000 HIS (Table 1).

#### 2.2.4. Estimation of the Excess Risk of Deaths Among People with Disability

The excess mortality risk associated with severe disability was estimated using survival models. Although the Cox proportional hazards model is widely regarded as the standard analytical approach, it relies on the assumption of proportional hazards across covariates. This assumption was evaluated using statistical tests and graphical diagnostics based on scaled Schoenfeld residuals [19], which indicated violations primarily due to time since baseline influencing risk. Consequently, we adopted a Weibull regression model, which provided a better fit and allowed greater flexibility. Hazard ratios were derived from interaction terms between age group and disability status, in models that also included the main effects of age group and disability.

#### 2.2.5. Building a Modified Life Tables Through the Excess of the Death Risk of Persons with Disabilities

Hazard ratios were then used to estimate the ratio of mortality risk for people with severe disabilities relative to all survey participants within each age class. These ratios were applied as multiplicative factors to age-specific death probabilities from the general population life tables to construct a life table for persons with disabilities, under the assumption that the study sample reflects the general population. To obtain the probabilities of death for each year of life, the ratios estimated for ten-year age groups were interpolated using an exponential spline function [1]. The resulting life expectancy estimates represent the expected remaining years of life for individuals assumed to remain either nondisabled or disabled for the rest of their lifetime.

#### 2.2.6. Sensitivity Analysis

We performed a sensitivity analysis to show how LE estimates may change under alternative transition parameter specifications. Following the method proposed by Rickayzen [15] and described in previous paragraphs, the model parameters were calibrated so that the resulting age- and sex-specific prevalence rates closely matched those observed in the HISs conducted in 2005 (middle years of the follow-up period) and 2012 (final year of the follow-up period) [20,21].

## 3. Results

A total of 5908 people with disabilities were identified in the study sample. Of these, 3694 (60%) died during the observation period (1999–2012). Across the entire survey population, 14,912 deaths were recorded, indicating that 11.6% of the population died during the observation period.

**Figure 1:**
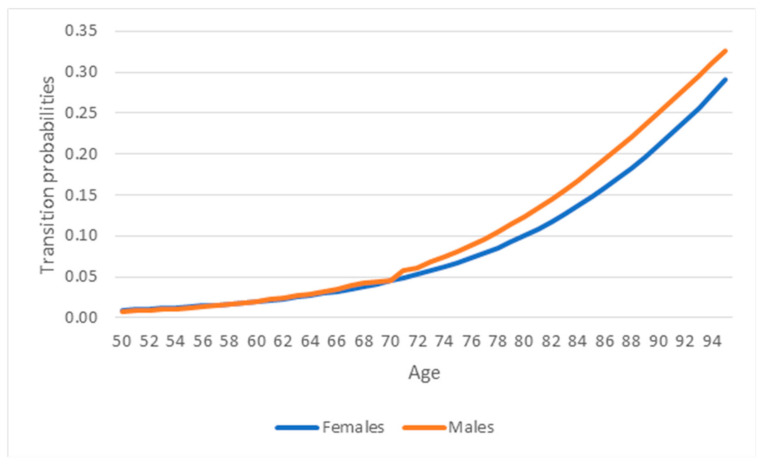
Transition probabilities from health status to disability status by age and sex.

Age-specific transition probabilities from a healthy state to a state of severe disability were estimated using the model proposed by Rickayzen [17]: the transition probabilities for females at age x were equal to A + (D − A)/(1 + BC − x) where the parameter A is the probability of being disabled at young ages; D is the probability of being disabled at extremely high ages; the parameters B and C determine how rapidly the probabilities change between the two extreme values; for males, a multiplicative correction term is added: 1-1/3 exp [−(x − E)/4)2], with the extra parameter, E, giving the age at which there is a “kink” in the transition probability function. The values of used parameters are as follows: (for males) A = 0.006, B = 1.106, C = 93.511, D = 0.452, E = 70.3002; (for females) A = 0.006, B = 1.093, C = 103.600, D = 0.613, E = not used. This model incorporates age-related prevalence patterns observed in HIS and provides a smoothed trajectory of transition risk over the life course. Parameters of the model were calibrated separately for males and females to closely reproduce observed disability prevalence in the baseline survey. The transition probabilities by age and sex are reported in Figure 1.

A longitudinal 13-year panel of subjects interviewed during 1999–2000 HIS was then reconstructed linking the date of the occurrence of deaths from the death registries 1999–2012 and attributing the disability condition both using the information collected with the HIS at the start of the observation and the annual transition probabilities to the health subjects in the following 13 years. Because severe disability was treated as an absorbing state, individuals who transitioned into disability were assumed to remain disabled for the remainder of the follow-up.

Mortality risks for individuals with and without disability were estimated using a Weibull survival model. The Cox proportional hazards model was initially evaluated but rejected due to violations of the proportional hazard assumption based on scaled Schoenfeld residual diagnostics. The Weibull model, which accommodates non-proportional hazards, provided a better fit to the observed data. Interaction terms between age class and disability status were used to derive age-specific hazard ratios. The hazard risks of the subject with disabilities compared to those without disabilities was therefore estimated with the following Weibull model H(t) = λ t^λ−1^ × exp[−λ [b_0_ + b_1_X_1_ + b_2_X_2_ + b_3_X_1_X_2_]], where X_1_, X_2_ represent the predictor variables (X_1_ = age-class, X_2_ = presence of disability) and λ is a shape parameter. The fitting of the model was tested using the linktest function of Stata 17 [22]. The risk ratio (RR) between the risk of dying among persons with disabilities, by ten-year age groups, and the risk of dying of all subjects participating in the 1999–2000 HIS was then calculated. The RR shows its highest value in the age group 25–34 years, decreases gradually in the next age groups, reaching values close to one over 75 years old. Estimation for the age class 6–14 was not represented because not estimable through the adopted Weibull model (Table 2).

**Figure 2:**
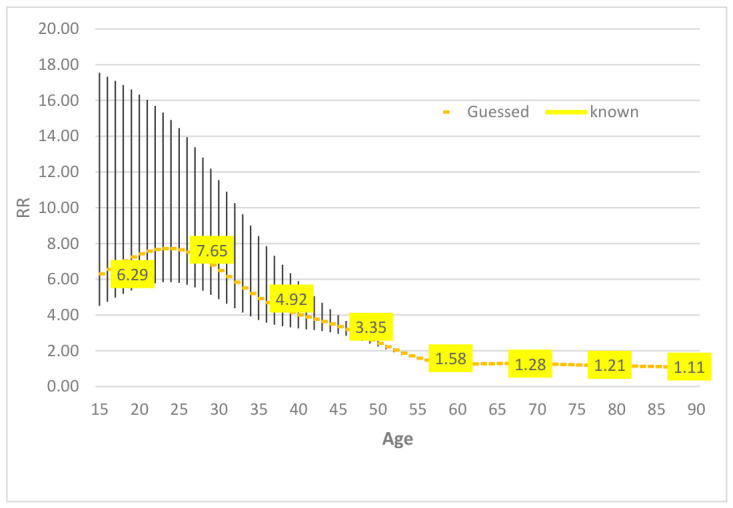
Interpolation of RRs with a spline cubic equation with 95% confidence bands.

Estimates of RRs for ten-year age classes were interpolated with spline cubic equation, yielding estimates for one-year age group (Figure 2).

The interpolated hazard ratios were applied as multiplicative adjustments to age-specific mortality probabilities from the 2012 general population life tables, producing separate life tables for males and females with severe disabilities. Because mortality data below age 15 were not reliable, life expectancy was calculated beginning at age 15. The resulting estimates reflect remaining life expectancy under the assumption that the individual remains in the same disability state throughout life.

**Figure 3:**
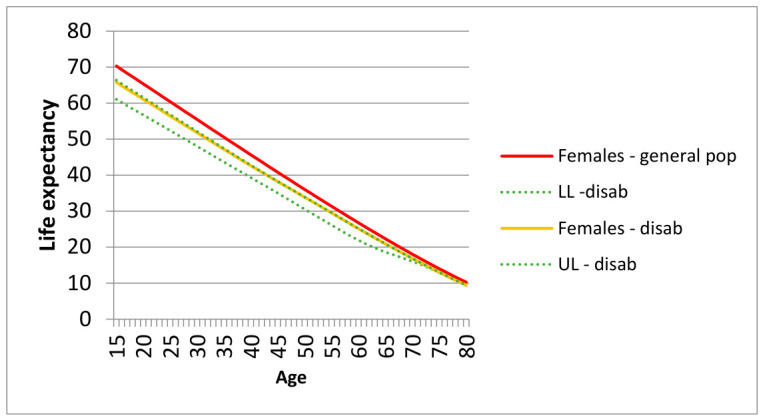
Life expectancy (LE) of general population and persons with disabilities, with 95% confidence intervals (LL = lower limits; UL = upper limits): Females.

**Figure 4:**
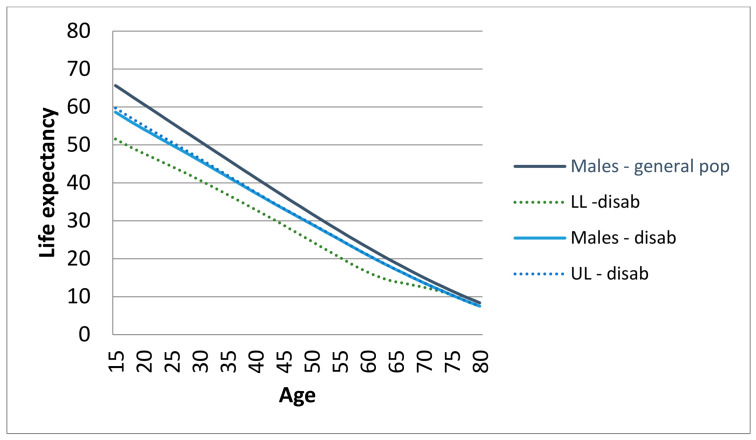
Life expectancy (LE) of general population and persons with disabilities, with 95% confidence intervals (LL = lower limits; UL = upper limits): Males.

The LE at age 15 for people with disabilities was 59.1 years for men and 66.2 years for women. The LE gap at 15 years between people with disabilities and the general population was 6.6 years for men and 4.1 years for women. LE at 30 years for people with disabilities was 46.4 years for men and 52.2 years for women, with gaps of 4.6 years and 3.3 years, respectively. As expected, the gap in LE tends to be lower at later ages: −1.9 years at 55 years, −1.5 years at 65 years, and −1.1 years at 75 years for men; and −1.5 years at 55 years, −1.2 years at 65 years, and −0.9 years at 75 years for women. Estimated LEs at various ages for people with disabilities, with associated confidence intervals, compared with those of the general population, are reported separately by sex in Figure 3 and Figure 4.

### Results of the Sensitivity Analysis

When the disability rates derived from the HIS 2005 were used as the reference for the model estimating transition probabilities, compared to the model that used the 1999 disability rates as its reference, the estimates of LE for people with disabilities were slightly lower. The differences ranged from a maximum of 0.56 years at age 30 to a minimum of 0.06 years at age 75 for males, and from a maximum of 0.42 years at age 30 to a minimum of 0.05 years at age 75 for females.

Furthermore, if the model used to estimate transition probabilities adopted disability rates derived from the 2012 Health Interview Survey (HIS) rather than the 1999 disability rates, the estimates of LE for people with disabilities were slightly lower. The differences ranged from a maximum of 0.54 years at age 30 to a minimum of 0.08 years at age 75 for men, and from a maximum of 0.40 years at age 30 to a minimum of 0.07 years at age 75 for women. Further details on the sensitivity analysis are available as Supplementary Materials.

## 4. Discussion

This study provides new evidence on the life expectancy of individuals with severe disabilities in Italy, drawing on data from the principal national sample survey on population health, which was linked to the national register of causes of death. At age 15, life expectancy was 6.6 years lower for men and 4.1 years lower for women compared with the general population. The risk ratio (RR) of death for persons with severe disabilities, compared with those without, ranged from 7.6 in the 25–34 age group to 1.1 in the 75+ age group. These results are broadly consistent with findings reported in the existing literature.

Strauss and colleagues [23] reported that individuals with no mobility capacity had a nearly fivefold increase in the risk of death at age 15 relative to peers with good mobility, whereas individuals with reduced mobility had twice the risk [24]. Similarly, Majer et al. [4], in a Dutch study, estimated life expectancy at age 55 among persons with disabilities to be 15.9 years for men and 21.3 years for women—values in line with those obtained in the present study, which provides a more comprehensive picture by estimating life expectancy from age 15 onwards. Life expectancy for people with physical disabilities in China is lower than for the general population, with a gap in life expectancy at birth of 17.1 years for males and 12.7 years for females [25].

A recent study investigated mortality and life expectancy among people with disabilities according to disability type in Korea, using combined data from 2008 to 2017. Life expectancy at birth for people with disabilities was found to be 65.2 years (95% CI: 64.9–65.5), with a 17.6-year gap between the disabled and non-disabled populations [26].

A recent systematic review estimated the mortality rate ratios of people with and without disabilities by age group. The pooled hazard ratio was estimated to be 4.46 (95% CI 3.01–6.59) for children aged 0–15 years, 3.53 (95% CI 1.29–9.66) for adults aged 15–59 years, and 1.97 (95% CI 1.63–2.38) for adults aged over 60 years [27]. These estimates are comparable with those produced in our study. A recent Italian study estimated that life expectancy with disabilities for people aged 50–79 years ranged from 8.1 to 12.6 years. These figures are difficult to compare as they concern a wide age range (50–79 years) and are not distinguished by sex. Nevertheless, the life expectancies of this study are like those estimated in our study for 79-year-olds.

Unlike the general population, the population surveyed in our study is free-living. Cambois [28] estimated the difference in life expectancy between free-living and institutionalized populations. In younger age groups, where disability is rarely prevalent, the difference in life expectancy between free-living and institutionalized people is estimated to be around seven months. As people get older and become more likely to experience disability, this difference decreases until it disappears.

In our study the OECD indicator was preferred to the Global Activity Limitation Indicator (GALI), whose validity to accurately categorize the degree of severity of an individual’s disability was not considered so high, due to its limited sensitivity [29]. For these reasons, the OECD indicator, which has been used for many years with reliable and objective validity in assessing the severity of disability, was instead used.

### Strengths and Limitations

One of this study’s main strengths is that, to our knowledge, it is the first to provide life expectancy estimates for people with disabilities in Italy using data from a national survey. It is also one of the few studies of its kind available worldwide. Another strength is that survival measures are estimated based on a large, representative survey of free-living individuals who were followed up for 13 years. Moreover, the LE estimates by age and sex are robust and not significantly influenced by alternative transition parameter specification.

However, this study has several limitations. Firstly, the disability data were self-reported, which can result in under- or overreporting of disability, thereby biasing the outcomes. Nevertheless, self-reported measurement of functional limitations has been shown to consistently reflect similar assessments of function [30].

As is usual, the probabilities of death were not calculated for a generation of contemporaries, but rather by observing subjects at follow-up entry for the subsequent 13 years. It is therefore assumed that the relationship between the risk of death among people with disabilities and the entire observed population remains constant throughout the observation period.

To address the issue that disability status was only ascertained at the start of the follow-up period, transition probabilities (TP) were used to predict changes in state from absence to presence of disability, categorized by age and sex. TP were estimated by assuming a stationary population structure through a function with parameters calibrated to closely replicate the age- and sex-specific prevalence rates of disability at entry. Both assumptions have a margin of uncertainty that cannot be tested, although large deviations from these assumptions are quite unlikely.

The assumption that severe disability is irreversible may not always be true, which can lead to an overestimation of the mortality risk among disabled people. However, it should be noted that, given the definition of severe disability under consideration, the possibility that the disability might be reversible is extremely remote.

The available database information does not allow for distinction between types of disability and their associated probabilities of death. This therefore prevents life expectancy estimates from being adjusted for specific disability conditions, for which relevant data are already available in the literature.

Furthermore, the validity of the current study assumes that disability rates in Italy have not changed significantly over the past twenty years. This can be verified using the data of the disability information system of the National Institute of Statistics [31], which shows that rates of severe disability in Italy have remained unchanged since 2009.

Finally, the data does not permit the calculation of accurate RRs for individuals below 15 years of age. Furthermore, many factors that interact with life expectancy could not be considered due to the limitations of the available data. The scientific literature has highlighted that each individual factor (immobility, incontinence, swallowing problems and the presence and level of intellectual disability) has a distinct negative impact on life expectancy. Therefore, it is reasonable to assume that life expectancy decreases with the presence of multiple factors [2].

Lastly, the linkage procedure between the survey population and death records was successful in 92% of cases. It is possible that there may be a selection bias in the remaining 8% of the study population, although this would have a limited impact on the results. However, health status is unlikely to affect the record linkage result because the key is based on sociodemographic variables.

## 5. Conclusions

This study provides robust and population-representative evidence on the life expectancy gap between individuals with and without disabilities, offering valuable insights for policy evaluation and planning. In Italy, 47.7% of people with disabilities under the age of 40 live with two parents, 15.3% live with a single parent and 9.5% live alone. These individuals are at risk of being left without assistance in the future. Therefore, it is essential to understand the life expectancy of people with disabilities for policy planning, particularly when designing interventions to support those who lose parental or spousal care.

People with disabilities are at risk of outliving their family members, particularly if their parents are elderly. In the future, these individuals may be at increased risk of exclusion and marginalization if society is unable to provide them with the care, support and economic autonomy that are currently mainly ensured by family networks in Italy. However, this approach may become less effective as family support is likely to diminish due to rapid demographic changes, which have resulted in family structures becoming older and having fewer children. Data on life expectancy can help ensure that adequate resources are allocated for the care of people with severe disabilities who outlive their family caregivers. Looking ahead, welfare systems will need to expand their provision of personal care services and co-housing solutions to address the growing number of elderly people, and consequently people living with disabilities.

Due to the lack of a registry for the population with disabilities or longitudinal studies on this group, estimating survival is highly complex. Without certain assumptions, it would have been impossible to produce estimates based on cross-sectional data. The resulting estimates indicate a low level of uncertainty, as evidenced by the confidence intervals associated with the point estimates. Future efforts to improve these estimates will aim to replicate the methodology using data from multiple health surveys and additional years of mortality data.

In conclusion, the life expectancy of a 15-year-old boy is 59.1 years, whereas for a 15-year-old girl it is 66.2 years. This is lower than the life expectancy in the general population, where the expected years of life at age 15 are 66.8 for men and 70.8 for women. Based on these estimates, a 15-year-old boy living with one or two 40-year-old parents, on average, would expect to spend 5 to 8 years without at least one parent. This aspect is important for planning policies for families with people with disabilities.

## Figures and Tables

**Table 1:** Number of subjects, number of deaths and person-years by disability status and age group. Observation period: 1999–2012.

	Persons with Disabilities			Persons Without Disabilities		
Age Class	Subjects	Deaths	Person-Years	Subjects	Deaths	Person-Years
6–14	156	0	2028	12,668	27	164,540
15–24	125	6	1576	16,569	84	214,880
25–34	151	11	1859	19,955	159	258,560
35–44	172	24	2096	20,420	386	263,613
45–54	275	54	3260	18,654	869	238,419
55–64	565	212	5932	15,060	1855	186,557
65–74	1253	721	11,734	12,234	3727	140,565
75–84	1841	1436	14,099	5406	3277	52,718
85+	1370	1230	7304	966	832	6913
Total	5908	3694	49,888	122,910	11,218	1,539,469

**Table 2:** Ratio of the risk of death (RR) of persons with disabilities compared that of all respondents in the 1999–2000 HIS, and 95% confidence intervals: lower limits (LL); upper limits (UL).

Age Class	RR	LL	UL
15–24	6.29	4.52	17.56
25–34	7.65	5.80	14.45
35–44	4.92	3.73	8.41
45–54	3.35	2.95	3.99
55–64	1.58	1.51	1.66
65–74	1.28	1.26	1.29
75–84	1.21	1.22	1.21
85+	1.11	1.13	1.10

## Data Availability

Data used in this study may be obtained from the Italian Health Interview Survey 1999–2000 (HIS) linked to the Italian Register of causes of death through 2012 and are not publicly available.

## Supplementary Materials

The following supporting information can be downloaded at: https://www.mdpi.com/article/10.3390/epidemiologia7030065/s1. Table S1: Rates per 100 of persons with severe disability by age classes in Italy. Surveys 2000, 2005, 2013; Table S2: Number of person-years of persons disability and disability rates per 100 by age group in cohort built according to transition probabilities estimated through the disability rates observed in HIS 1999, 2005 and 2012. Observation period: 1999–2012; Table S3: Ratio of the risk of death (RR) of persons with disabilities compared that of all respondents, and 95% confidence intervals: lower limits (LL) upper limits (UL) considering the transition probabilities based on the 2005 HIS survey; Table S4: Ratio of the risk of death (RR) of persons with disabilities compared that of all respondents, and 95% confidence intervals: lower limits (LL) upper limits (UL) considering the transition probabilities based on the 2012 HIS survey; Table S5: Estimation of LE at age 15, 30, 45, 55, 65 and 75, by sex, in the general population and among people with disabilities based on the transition probabilities derived respectively from the HIS 1999, 2005 and 2012.

## Abbreviations

The following abbreviations are used in this manuscript:

ADL	Activities Daily Living
CI	Confidence Intervals
HIS	Health Interview Survey
HR	Hazard Ration
OECD	Organization for Economic Co-operation and Development
RR	Risk Ratio
TP	Transition probabilities
