# Supplementary material for: Life Expectancy of Persons with Disability in Italy: Estimation Based on an Administrative Cohort from 1999 to 2012
Source: Epidemiologia (Basel). 2026 May 7;7(3):65. doi: 10.3390/epidemiologia7030065 (PMC13214677; doi:10.3390/epidemiologia7030065)
Supplement: Supplementary file 1 [file epidemiologia-07-00065-s001.zip › epidemiologia-4163961-supplementary.pdf]

Supplementary material

Sensitivity analysis.

As life expectancy estimates may change under alternative specifications of transition parameters, the model parameters were calibrated so that the resulting age- and sex-specific disability rates closely matched those observed in Health Interview Survey (HIS) conducted in 2005, years included in the middle of the follow-up period, or those observed in the HIS conducted in 2012, final years of the follow-up period, in addition to the year 1999, which is the year considered in the study

Table S1 shows the prevalence of severe disabilities observed in Italy in surveys conducted in 1999, 2005, and 2012. It is noted that the age-specific disability rates are similar across the three surveys

Age-specific transition probabilities from a healthy state to a state of severe disability also in the sensitivity analysis were estimated using the model proposed by Rickayzen. The model includes one parameter (A) that is the probability of being disabled at young ages; and the parameter D which is the probability of being disabled at extremely high ages. In the sensitivity analysis the model was replicated adopting the new parameters (A) and (D) derived respectively by the data of the 2005 and 2012 HIS surveys.

Table S2 reports the number of person-years of persons disability and disability rates per 100 by age group in cohorts built according to the transition probabilities estimated through the disability rates observed in the HIS held in 1999,2005 and 2012.

The ratios of the risk of death (RR) of persons with disabilities compared that of all respondents were recalculated considering the two different transition probabilities from the health status to severe disability status along the 13-year follow-up period derived respectively from the 2005 HIS survey (table S3) and from the 2012 HIS survey (Table S4)

The LE estimates of people with disabilities based respectively on the transition probabilities derived from the 1999,2005 and 2012 HIS surveys are reported in table S5. Estimates based on different scenarios show very similar values, with a slight decrease between 1999 and 2005 and substantial stability between 2005 and 2012. The absence of significant changes over time indicates that the differences observed by age and sex are robust and not significantly influenced by alternative transition parameter specifications.

Table S1. Rates per 100 of persons with severe disability by age classes in Italy. Surveys 2000, 2005, 2013

Year of the survey	Age classes										
	6-14 years	15-24 years	25-34 years	35-44 years	45-54 years	55-64 years	65-69 years	70-74 years	75-79 years	80+ years	Total
2000	1.60	0.84	0.85	1.04	1.56	3.61	7.00	11.47	21.16	43.89	4.87
2005	1.63	0.59	0.61	0.91	1.31	2.47	5.36	9.69	17.74	41.65	4.73
2012	1.60	0.69	0.70	1.18	1.48	2.88	5.20	9.36	18.60	42.75	5.62

Table S2. Number of person-years of persons disability and disability rates per 100 by age group in cohort built according to transition probabilities estimated through the disability rates observed in HIS 1999,2005 and 2012. Observation period:1999-2012

	**Persons with disabilities (HIS 1999)**		**Persons with disabilities (HIS 2005)**		**Persons with disabilities (HIS 2012)**	
**Age class**	**person-years**	**Rate**	**person-years**	**Rate**	**person-years**	**Rate**
15-24	1,723	0.94	1,723	0.94	1,723	0.94
25-34	1,702	0.76	1,702	0.76	1,702	0.76
35-44	1,988	0.77	1988	0.77	1988	0.77
45-54	6,055	2.46	5,765	2.35	5,934	2.42
55-64	24,121	11.29	21925	10.27	22540	10.55
65-74	40,706	22.28	36,342	19.89	36,170	19.80
75-84	52,105	42.63	47,094	38.53	46,200	37.80
85+	29,895	71.88	28,034	67.41	27,505	66.14
Total	159,233	10.02	144,573	9.15	143,762	9.10

Table S3. Ratio of the risk of death (RR) of persons with disabilities compared that of all respondents, and 95% confidence intervals: lower limits (LL) upper limits (UL) considering the transition probabilities based on the 2005 HIS survey

Age class	RR	LL	UL
15-24	6.23	4.48	17.19
25-34	7.58	5.75	14.23
35-44	4.88	3.70	8.32
45-54	3.59	3.20	4.19
55-64	1.60	1.53	1.69
65-74	1.44	1.42	1.46
75-84	1.22	1.22	1.22
85+	1.12	1.11	1.13

Table S4. Ratio of the risk of death (RR) of persons with disabilities compared that of all respondents, and 95% confidence intervals: lower limits (LL) upper limits (UL) considering the transition probabilities based on the 2012 HIS survey

Age class	RR	LL	UL
15-24	6.23	4.48	17.20
25-34	7.58	5.75	14.24
35-44	4.88	3.70	8.32
45-54	3.50	3.12	4.09
55-64	1.58	1.51	1.66
65-74	1.45	1.43	1.47
75-84	1.23	1.22	1.23
85+	1.12	1.11	1.13

Table S5. Estimation of LE at age 15, 30,45,55,65 and 75, by sex, in the general population and among people with disabilities based on the transition probabilities derived respectively from the HIS 1999,2005 and 2012

	**General population**		**People with disab. (HIS 1999)**		**People with disab. (HIS 2005)**		**People with disab. (HIS 2012)**	
Age	Males	Females	Males	Females	Males	Females	Males	Females
15	65.7	70.3	59.1	66.2	58.6	65.8	58.7	65.8
30	51.0	55.5	46.4	52.2	45.9	51.8	45.9	51.8
45	36.6	40.8	33.7	38.6	33.2	38.2	33.2	38.2
55	27.4	31.3	25.5	29.8	25.0	29.5	25.0	29.5
65	18.9	22.3	17.4	21.1	17.2	20.9	17.2	20.9
75	11.6	14.0	10.5	13.1	10.5	13.0	10.4	13.0
